# Evaluating Interface Pressure Distribution and Range of Motion in a Mesh-Structure 3D-Printed Lumbar Brace

**DOI:** 10.3390/bioengineering13080910

**Published:** 2026-08-12

**Authors:** Hsiang-Chieh Hsu, Po-Yin Chen, Chen-Sheng Chen

**Affiliations:** Department of Physical Therapy and Assistive Technology, National Yang Ming Chiao Tung University, Taipei 112304, Taiwan; hchhh427@gmail.com (H.-C.H.); poyin1128@nycu.edu.tw (P.-Y.C.)

**Keywords:** spinal braces, 3D printed, pressure distribution, ROM, biomechanics

## Abstract

Conventional spinal braces often suffer from poor fit and low breathability, with most 3D-printing research focusing on scoliosis rather than general support. This study evaluated the biomechanical performance and range of motion (ROM) restriction of a semi-custom 3D-printed brace (3DP) compared with two traditional braces (Medi-H, Medi-S). Sixteen participants performed static standing, flexion, extension, and pick-up tasks while wearing each brace. Interface pressure was recorded across seven anatomical regions, and thoracic (C7–T8) and lumbar (T8–L3) ROM were assessed simultaneously using repeated-measures ANOVA. Results showed that 3DP and Medi-H provided significantly greater overall pressures than Medi-S. During flexion and pick-up tasks, 3DP delivered enhanced support to the middle lumbar region and erector spinae, whereas Medi-H concentrated greater pressure on the spine, particularly during extension. Thoracic and lumbar ROM were restricted comparably across all braces (20–30°). In conclusion, 3DP achieves an equivalent restriction of sagittal motion to traditional braces while offering superior regional support to the upper and middle lumbar regions.

## 1. Introduction

Spinal braces are widely used in rehabilitation settings to restrict excessive trunk motion, protect the spine from injury, and enhance postural stability during daily activities. Modern lifestyle patterns, such as prolonged sitting and repetitive trunk movements, frequently impose repetitive mechanical loading on the lumbar spine, which may contribute to low back discomfort, impaired postural control, and clinically diagnosed pathology [1]. Consequently, previous studies have demonstrated that appropriate external spinal support can reduce pain and improve motor control, making spinal braces a valuable conservative intervention [2,3,4].

Spinal braces are commonly prescribed for postoperative rehabilitation following lumbar surgery and for individuals with subacute low back pain as part of conservative management [5,6,7,8]. Recent studies have reported postoperative bracing rates following lumbar surgery ranging from 26% to 38%, reflecting the continued clinical application of bracing in selected patient populations [5,6]. Despite their widespread clinical use, most commercially available spinal braces are manufactured in standardized sizes, which may not adequately accommodate individual variations. This often results in suboptimal fit and reduced comfort, ultimately leading to decreased user compliance [9].

The mechanical rationale of spinal braces generally falls into two primary design strategies [10]. The primary objective is to increase intra-abdominal pressure by providing circumferential compression, thereby enhancing spinal stability through increased core muscle activation [11]. Braces that adopt this approach are typically fabric-based and lighter but may cause heat buildup during prolonged wear. The second objective involves directly restricting trunk motion through their structural rigidity [3]. Although rigid braces provide more effective restriction of the spinal range of motion, their bulkier profile and limited flexibility might compromise comfort and compatibility with movement. As a result, finding an optimal balance between support, mobility, breathability, and user comfort remains a challenge in spinal brace designs.

To address the limitations of traditional braces, customized spinal braces have become increasingly popular in recent years to meet the needs of specific groups. Enabled by advancements in 3D body scanning, computer-aided design, and manufacturing technologies, these braces can be tailored to every individual’s torso geometry and their requirements. Customized braces have been shown to increase adherence, maintain better spinal curves, and decrease manufacturing time and cost compared to traditional designs [12,13].

Recent advances in manufacturing have enabled the development of fully customized 3D-printed spinal braces that provide improved conformity and personalized support. However, most published studies have focused on patients with scoliosis, where the primary objective is spinal curve correction [12,14,15,16]. Comparatively fewer studies have investigated 3D-printed spinal braces for non-scoliotic lumbar conditions. Existing studies in these populations have primarily evaluated conventional spinal braces by their range of motion restriction, and other important biomechanical characteristics remain largely unexplored [11,17,18,19]. In addition, a customized 3D-printed spinal brace typically requires three-dimensional body scanning and computer-aided design, making the fabrication process labor-intensive, time-consuming, and costly [12,13]. Therefore, to reduce these costs, there is a need to develop a semi-custom-made 3D-printed brace.

Given these gaps, this study aimed to evaluate the biomechanical characteristics of a semi-custom-made 3D-printed spinal brace compared with two conventional braces commonly used in clinical practice. By examining regional interface pressure and spinal angles across various functional tasks, this study aimed to determine whether a semi-customized 3D-printed brace might offer advantages in support distribution and motion control. The findings are expected to provide essential biomechanical evidence that may guide clinical decisions and inform the future development of a semi-customized 3D-printed spinal brace. The novelty of our research lies in two aspects: First, the study aimed to integrate a semi-customized 3D-printing brace with interface pressure mapping and IMU (Inertial Measurement Unit) spatial kinematic tracking across multi-directional functional tasks. Second, the study established a baseline biomechanical benchmark evaluating regional pressure distribution and trunk motion control of a semi-customized brace relative to conventional rigid and soft braces.

## 2. Materials and Methods

### 2.1. Spinal Braces

**Figure 1:**
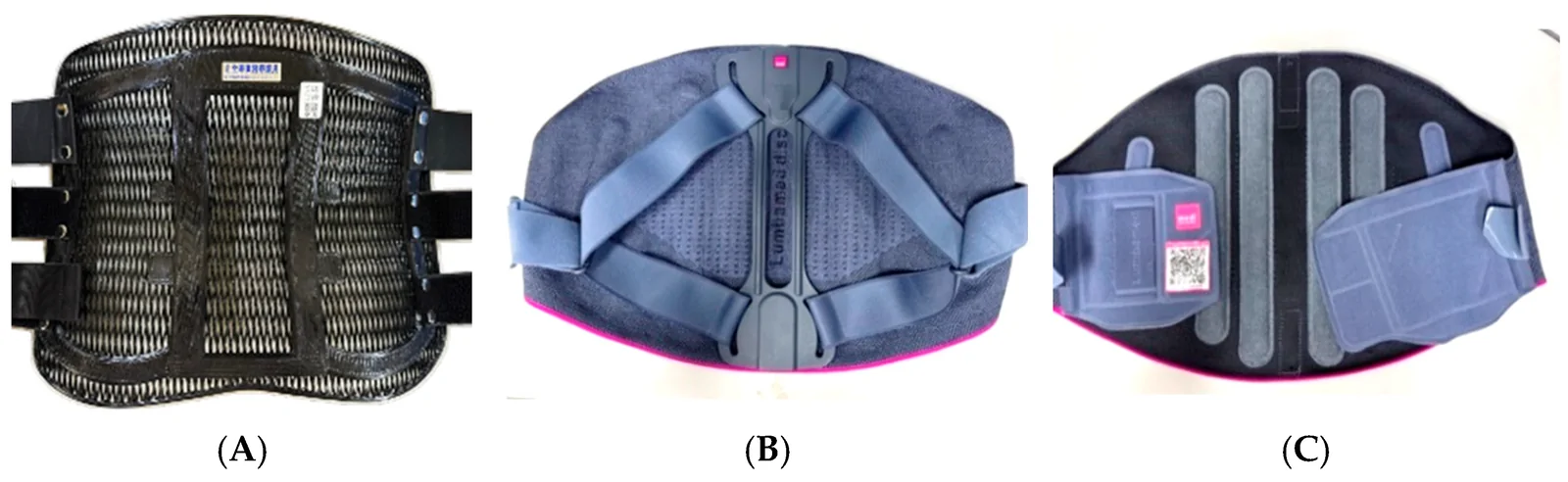
Spinal braces used in the study: (**A**) 3DP. (**B**) Medi-H. (**C**) Medi-S.

**Figure 2:**
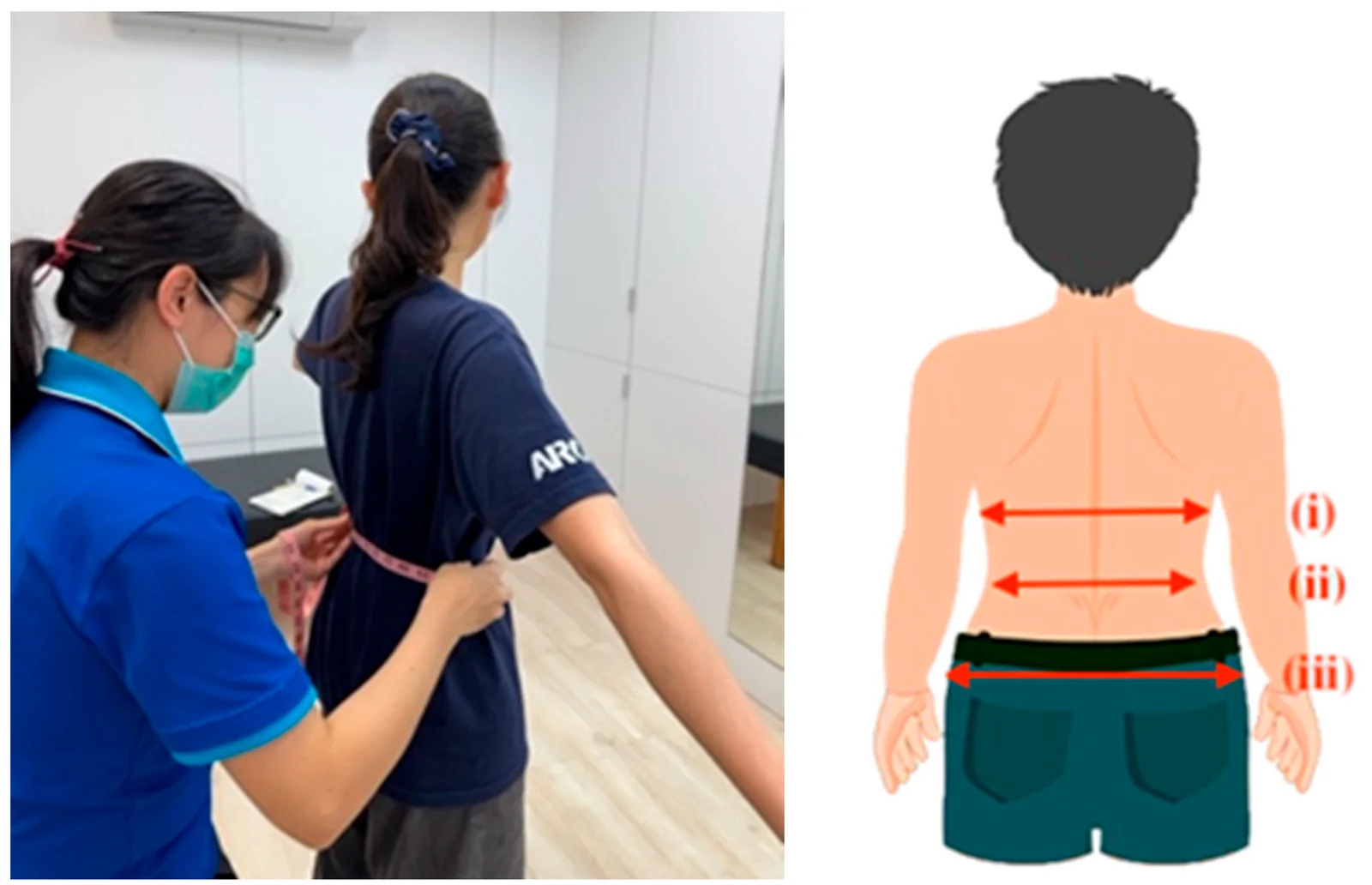
Semi-custom-made 3DP measurement. (i) chest circumference: measured at the lower chest line, (ii) waist circumference: measured at the narrowest part of the waist, and (iii) hip circumference: measured midway between the anterior superior iliac spine (ASIS) and greater trochanter.

Three types of spinal braces were chosen: a semi-customized 3D-printed brace (3DP; Cheng-Chuan Prosthetics & Orthotics Co., Taipei, Taiwan), a relatively traditional rigid spinal brace (Medi-H; Lumbamed^®^ disc, Medi GmbH & Co. KG, Bayreuth, Germany), and a relatively soft brace (Medi-S; Lumbamed^®^ disc, Medi GmbH & Co. KG, Bayreuth, Germany) (Figure 1). Traditional spinal braces have the disadvantage of creating a feeling of insecurity between the rigid support columns and the soft fabric, while the surrounding soft fabric provides insufficient support. The newly designed 3DP brace features three key characteristics: (1) its semi-rigid mesh structure provides more coverage compared to fabric; (2) its hollow mesh structure offers greater breathability; and (3) the rigid columns of the main structure are positioned on either side of the spine to assist in supporting the lumbar spine. Therefore, compared to traditional commercial braces, the 3DP brace offers greater breathability, coverage, and support. Additionally, the 3DP brace was fabricated semi-customized, as the subject’s trunk circumference was measured (Figure 2).

A fully customized brace relies on 3D body surface scanning and personalized slicing/printing. While individualized, it is labor-intensive, time-consuming, and cost-prohibitive for fabrication of a spinal brace. The 3DP brace evaluated here is more precisely described as semi-customized, reflecting its size selection process based on individual body measurements (chest, waist, and hip circumferences) rather than full patient-specific geometric reconstruction. The average weights of the three types of spinal brace (3DP, Medi-H, and Medi-S) were 603 g, 630 g, and 498 g, respectively.

All three braces focus on increasing contact pressure, while 3DP and Medi-H also have more direct rigidity limitations related to their material. 3DP is fabricated from thermoplastic polyurethane (TPU) with a breathable grid-structure design, known for its outstanding elasticity and stretchability. The Medi-H was covered with fabric, with metal straps inserted, and an external plastic panel that provides greater rigidity. In contrast, the Medi-S only relied on fabric coverage.

### 2.2. Participants

Sixteen healthy adults (13 females and 3 males; mean age 23 years; BMI 21 kg/m^2^; mean waistline 70.9 cm) voluntarily participated in this study. Participants were recruited through sampling from the local community. The inclusion criteria were: (1) age above 18-year-old; (2) no history of spinal surgery; (3) no current low back pain or musculoskeletal conditions affecting trunk movement; (4) body mass index within the normal range according to the World Health Organization classification (BMI 18.5–24 kg/m^2^); (5) waist circumference within the fitting range of all three spinal braces; and (6) the ability to independently perform all experimental tasks. Exclusion criteria included a history of spinal disease, recent low back pain, and anthropometric values outside World Health Organization (WHO) standards (BMI 18.5–24 kg/m^2^; waist circumference ≤ 90 cm for men and ≤80 cm for women).

This study was approved by the Institutional Review Board of National Yang Ming Chiao Tung University (NYCU 113149AE). The participant provided informed consent prior to the experiment.

### 2.3. Contact Pressure Measurement

A pressure sensor mat (Tactilus^®^ Seat Pressure Mapping System, Sensor Products International Srl., Bologna, Italy) was placed between the braces and the individual’s torso to record pressures. The pressure sensors used in this study (operating within a target measurement range of 0–5 PSI/0–0.35 kg/cm^2^) demonstrated an accuracy of ±10% and a precision of ±2%, with a sampling frequency of 100 Hz.

Before data collection, participants stood in a relaxed upright posture while wearing the spinal brace. Palpation was performed through the superior and inferior borders of the brace, the lateral region of the brace, and the anatomical landmarks (spinal processes and erector spinae). The corresponding pressure responses were displayed on the pressure map system to identify these landmarks and define each anatomical boundary, ensuring that the pressure measurements were anatomically aligned for each participant throughout the experiment. The pressure mapping system was then zeroed to eliminate baseline pressure from clothing and brace application before pressure data were recorded during functional tasks.

**Figure 3:**
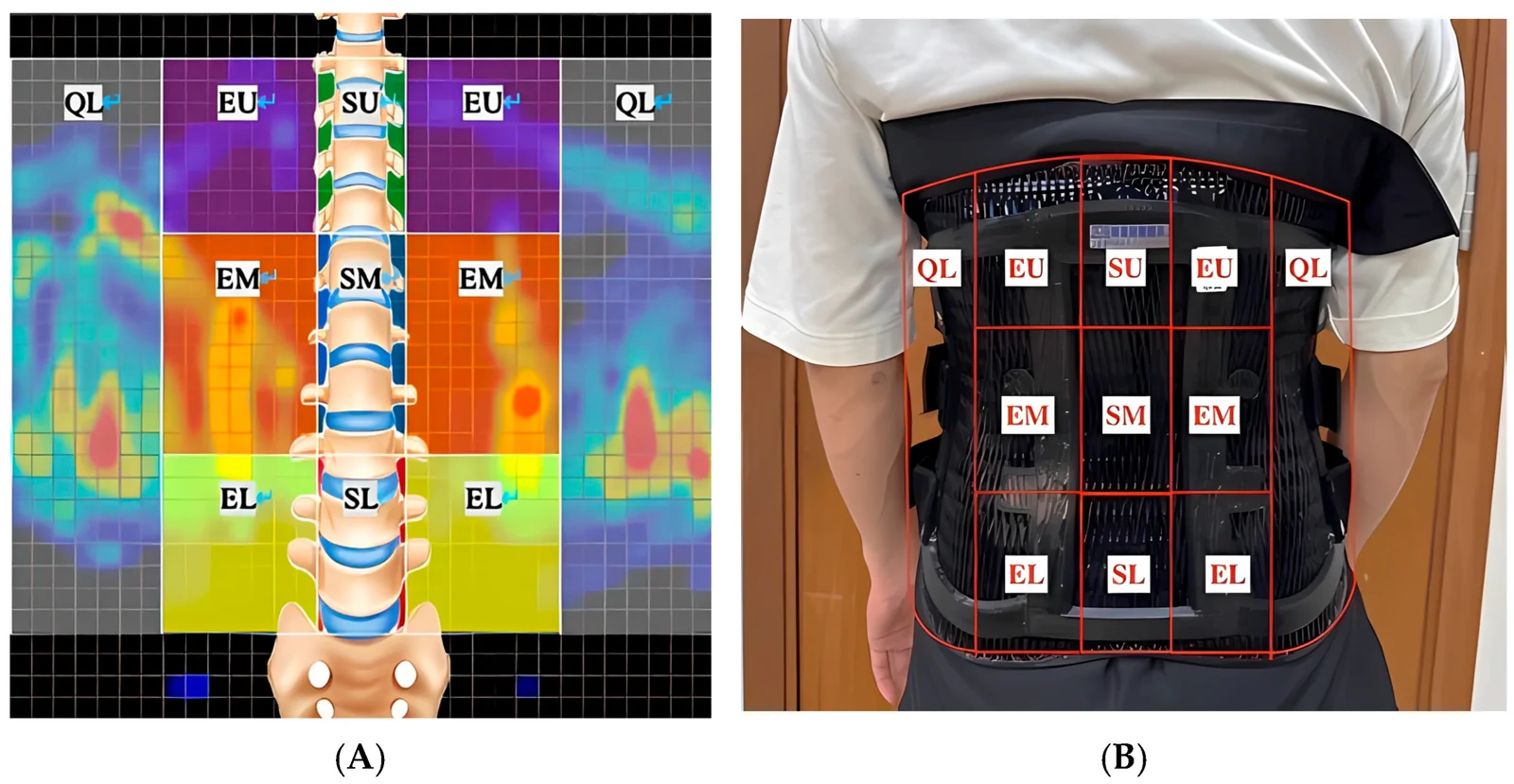
Pressure pad regions (**A**) mapping system diagram. The blue arrow indicate their locations. The colors are variation of pressures. (**B**) actual wearing diagram. Note: 1. SU: upper spine region; 2. SM: middle spine region; 3. SL: lower spine region; 4. EU: bilateral upper erector spinae region; 5. EM: bilateral middle erector spinae regions; 6. EL: bilateral lower erector spinae regions; 7. QL: bilateral sides of the quadratus lumborum.

Interface pressure was evaluated across three primary functional zones of the brace: the upper region (upper thoracic area), the middle region (lumbar/lordotic area), and the lower region (sacral area). These regions collectively cover the posterior trunk, directly interfacing with the spinal segments and the underlying erector spinae musculature. To clarify the different locations, seven predefined anatomical regions were identified for pressure analysis: the upper, middle, and lower regions over the spinous processes (SU, SM, and SL), the corresponding erector spinae regions (EU, EM, and EL), and the bilateral quadratus lumborum region (QL) (Figure 3).

### 2.4. Motion Analysis

Recent validation studies have demonstrated that Xsens DOT IMUs provide clinically acceptable concurrent validity and reliability for assessing spinal ROM compared with gold-standard optical motion capture systems, supporting their application in spinal biomechanical research [20,21]. Therefore, three inertial measurement units (IMUs, DOT, Xsens, Enschede, The Netherlands) were placed at the spinous processes of C7, T8, and L3. This 3-sensor configuration allows simultaneous tracking of thoracic vs lumbar movement, which is critical since spinal braces intended for the lumbar region can cause compensatory thoracic motion.

IMU synchronization, heading reset, and orientation estimation were implemented using the Xsens DOT Software Development Kit (version 2023.6.0). Sensor orientation was obtained and exported directly as three-axis Euler angles using the manufacturer’s local coordinate system. Participants maintained a relaxed upright standing posture to initialize the IMUs and establish the reference orientation. The baseline Euler angles were zeroed in this neutral standing posture, and subsequent trunk motions were calculated relative to this reference. Sagittal plane motion (flexion/extension) was extracted from the exported Euler angle data to calculate the relative thoracic (C7–T8) and lumbar (T8–L3) range of motion (ROM). The local coordinate systems were defined relative to the neutral standing reference frame. Trunk joint kinematics were calculated using a ZYX Euler angle sequence, which is appropriate for trunk biomechanics within functional ROM (Z-axis: axial rotation; Y-axis: sagittal plane motion; X-axis: frontal plane motion).

Thoracic ROM (∆θ_thoracic) was defined as the change in sagittal plane angle between the C7 and T8 IMU, whereas lumbar ROM (∆θ_lumbar) was between T8 and L3. The formulas were as follows:

∆θ_thoracic = (θ_C7 − θ_T8)

∆θ_lumbar = (θ_T8 − θ_L3)

### 2.5. Procedures

All participants performed the four tasks—static standing, trunk flexion, trunk extension, and pick-up—which were selected as they represent fundamental Activities of Daily Living (ADLs) that challenge lumbar biomechanics in different sagittal planes:

1. Static Standing: Establishes baseline postural contact and lordotic support. The first 5 s of the upright position before starting all tasks
2. Trunk Flexion & Pick-up: Induces significant anterior shear forces and maximum intervertebral disc loading at L3–L4 [11]. Forward bending to the maximum comfortable range
3. Trunk Extension: Challenges posterior paraspinal loading and contact pressures at the superior/inferior brace margins [22]. Backward leaning to the maximum comfortable range.

For each condition, pressure distribution and IMU data were recorded simultaneously. All movements were measured three times consecutively, with speed controlled by the subject.

### 2.6. Data Analysis

Repeated-measures ANOVA was used to assess the effects of braces on all pressure and ROM, using IBM SPSS Statistics version 29.0.1.1. All statistical data are presented as means with standard deviations. Statistical significance was set at *p* < 0.05. If the main effect is significant (*p* < 0.05), a Bonferroni post-hoc test will be used to perform pairwise comparisons.

## 3. Results

### 3.1. ROM Data

Overall, there were no significant differences in thoracic (C7-T8) and lumbar (T8-L3) ROM between the three braces in all tasks (Table 1). While the flexion task was most restricted by 3DP (29.64°), the extension task was most restricted by Medi-H (−22.59°), followed by the pick-up task, which was restricted by Medi-S (23.47°) and 3DP (24.16°).

### 3.2. Regional Average Pressure

#### 3.2.1. Static Standing

**Figure 4:**
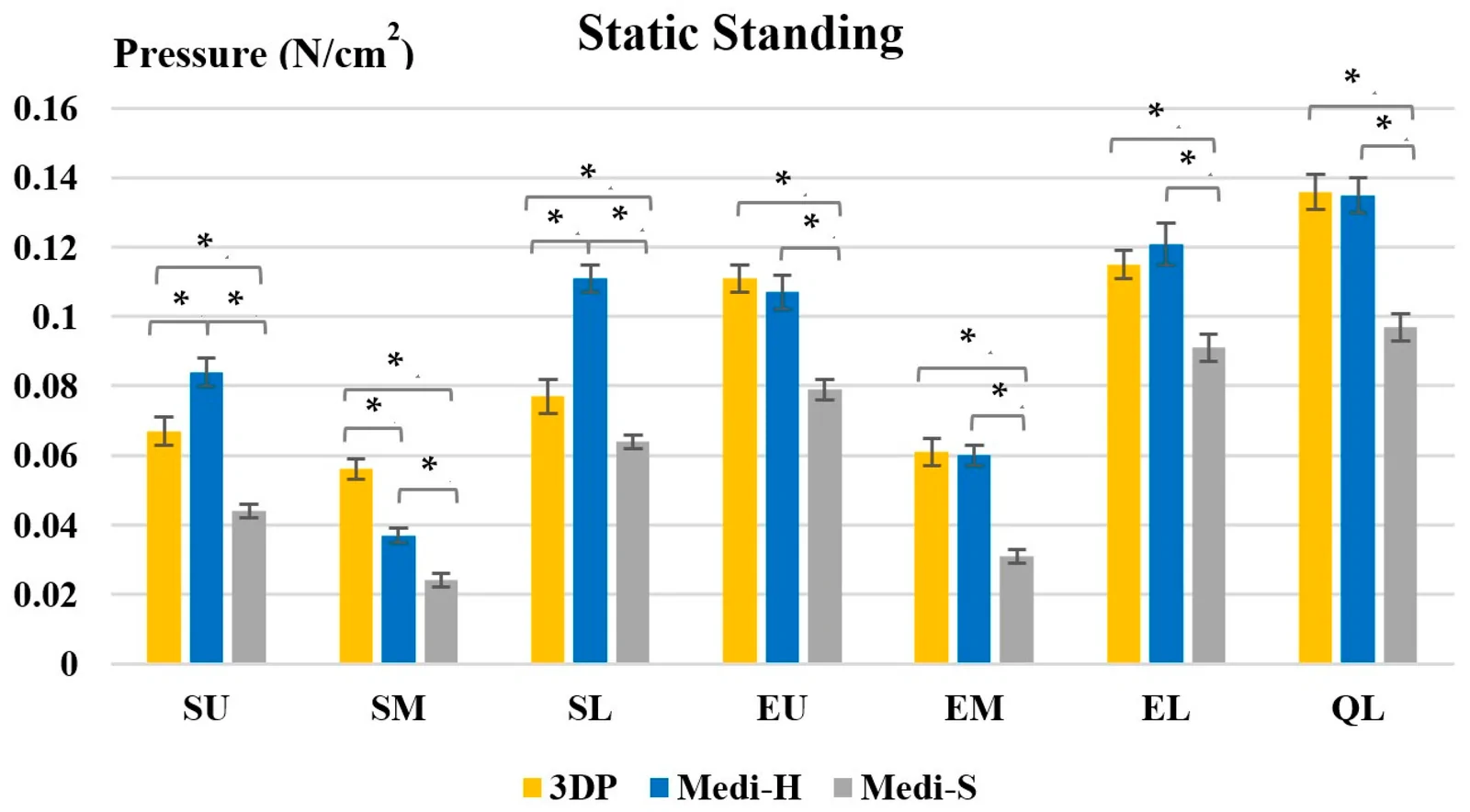
Pressure distribution in static standing. Note: * represents a significant difference (*p* < 0.05).

Significant differences across the spinal regions (SU, SM, and SL) were observed between the one brace and each other (Figure 4). The greatest pressure at SM was produced by 3DP (0.056 N/cm^2^), approximately 51% greater than Medi-H. While the greatest pressures at SU and SL were generated by Medi-H (0.084 and 0.111 N/cm^2^), over 25% greater than 3DP.

#### 3.2.2. Flexion

**Figure 5:**
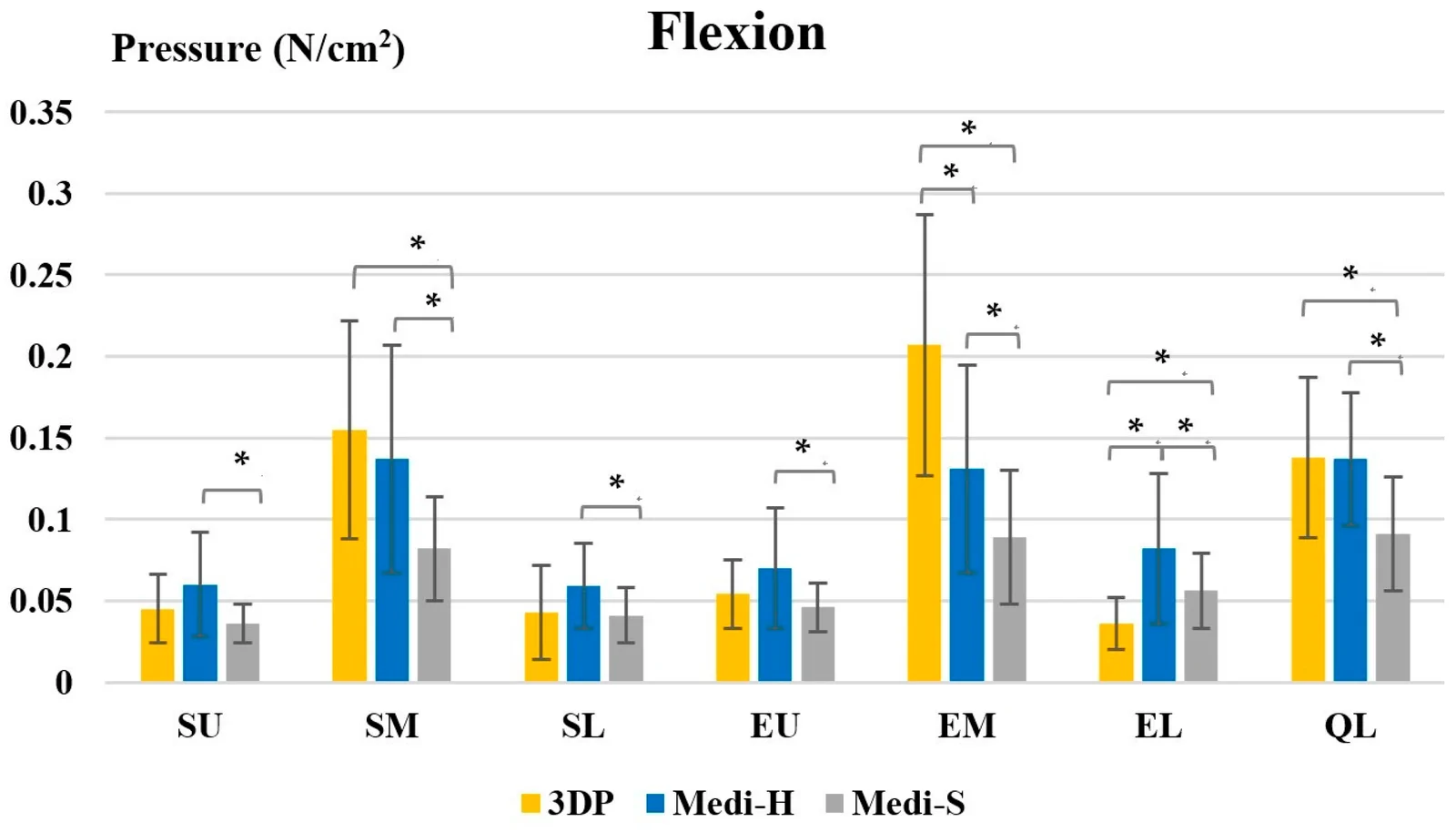
Pressure distribution in the flexion task. * represents a significant difference (*p* < 0.05).

All braces concentrated the pressure at the middle regions (SM and EM). 3DP exhibited significantly greatest pressure at EM region (0.207 N/cm^2^) than the Medi-H (0.131 N/cm^2^) and Medi-S (0.089 N/cm^2^). In contrast, 3DP demonstrated the least pressure at the EL region (0.036 N/cm^2^), whereas Medi-H exerted the greatest pressure (0.082 N/cm^2^) (Figure 5).

#### 3.2.3. Extension

**Figure 6:**
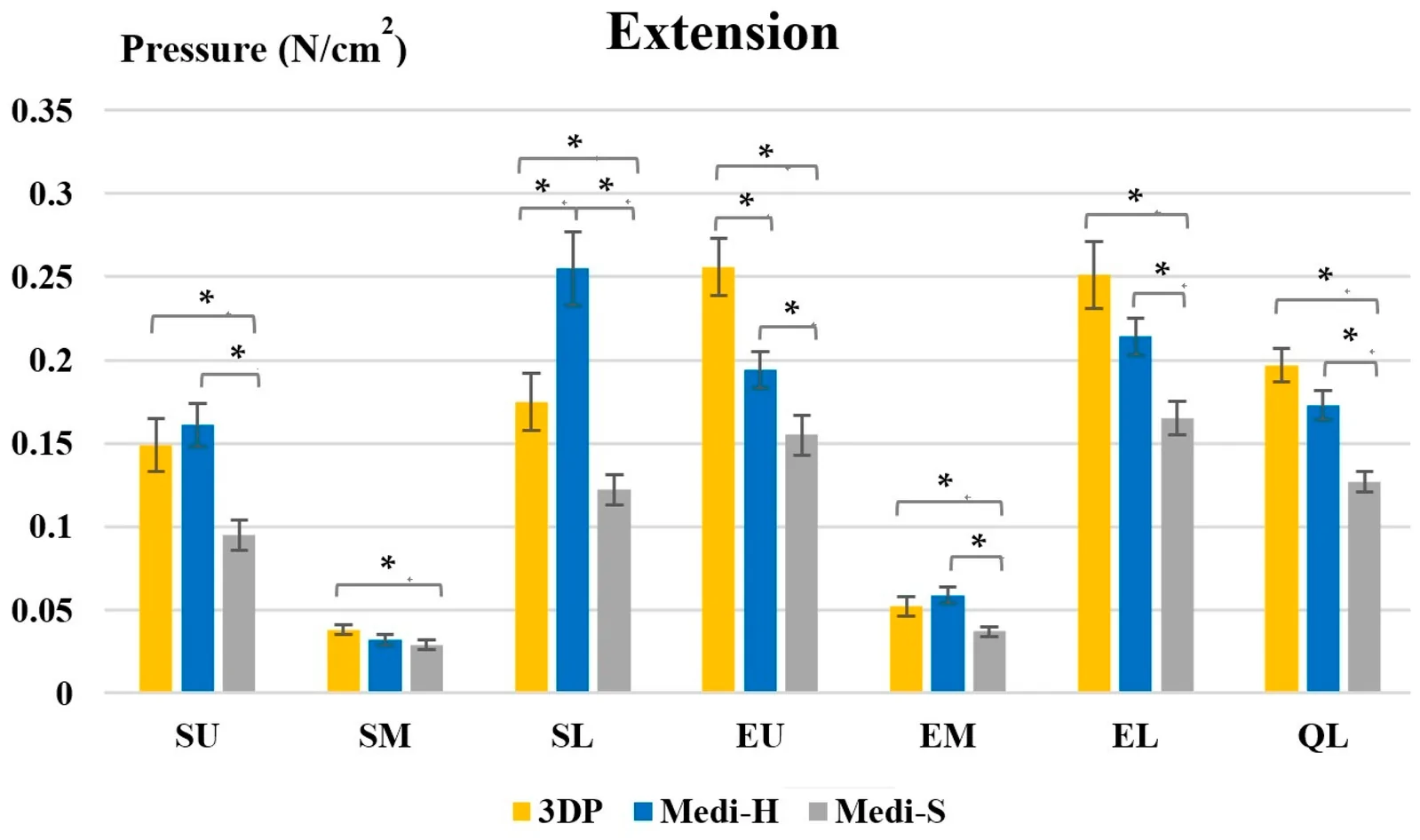
Pressure distribution in the extension task. * represents a significant difference (*p* < 0.05).

Pressures were generally elevated at the upper and lower regions. At the same time, Medi-H produced the greatest values in the spine region (SU and SL), with SL exceeding 0.25 N/cm^2^ and being significantly greater than those of the others. In contrast, 3DP showed the greatest pressures in the erector spinae region (EU and EL), with EU (0.256 N/cm^2^) significantly greater than Medi-H (0.194 N/cm^2^) and Medi-S (0.155 N/cm^2^) (Figure 6).

#### 3.2.4. Pick-Up

The distribution was similar to the flexion task, with pressures focused on SM and EM, but all slightly less. 3DP maintained the greatest EM value of 0.156 N/cm^2^, significantly greater than Medi-H (0.123 N/cm^2^) and Medi-S (0.073 N/cm^2^). The same pattern observed at EL was also evident at Medi-H, where the greatest pressure was recorded (0.091 N/cm^2^), while 3DP had the least pressure (0.039 N/cm^2^).

## 4. Discussion

Although spinal braces are widely used in conservative and postoperative management, the biomechanical mechanisms underlying their supportive function remain incompletely understood. A better understanding of pressure distribution is clinically important because it reflects how interface pressures are transferred from the brace to the individual and may influence both biomechanical performance. Therefore, our study aimed to compare the interface pressure distribution and trunk ROM among a semi-customized 3D-printed spinal brace, a relatively rigid brace (Medi-H), and a relatively soft brace (Medi-S) during functional daily activities.

### 4.1. Supportive Force Across Braces

In this study, we considered that pressures have different implications for trunk support at upper, middle, and lower regions. Overall, the greater interface pressures recorded in the upper and middle sensor regions of the brace indicated that these areas were the primary contact zones for transferring supportive forces to the trunk. The upper-region pressure can be considered the “upward support force” provided by braces, intended to support an individual’s trunk and reduce spinal intervertebral disc load [22]. As the middle region overlays the apex of the lumbar lordotic curve, the interface pressure generated in this region played a key role in maintaining lumbar curvature and providing proprioceptive feedback, which together contributed significantly to postural stability [23]. As for the lower region, its primary function was to engage the sacrum and pelvis, providing a stable anchoring foundation for the brace on the torso; consequently, it exerted a relatively limited direct influence on lumbar segmental motion compared with the upper and middle regions [24]. In addition, this study interpreted greater interface pressure as reflecting increased mechanical support provided by the brace. Furthermore, the maximum interface pressure recorded in this study was 0.256 N/cm^2^, substantially less than the commonly cited pressure-induced soft tissue injury threshold of approximately 32 mmHg (0.42 N/cm^2^) [25].

In the results, wearing 3DP demonstrated a more even pressure distribution than the other two traditional braces in static standing, reflecting its superior conformity and supportive force on the torso. Notably, the greatest peak pressures in the upper and middle regions (SU, SM, EU, and EM) were also predominantly observed with 3DP, suggesting a more effective supportive function in these essential regions. This finding is particularly meaningful when performing a forward movement task, as the middle region aligned with the L3–L4 segment, which experienced the greatest shear force during trunk flexion [26]. Therefore, the greater pressure observed in the middle regions (EM and SM) with the 3DP brace may indicate a more important centralized pressure distribution during the flexion and pick-up tasks, which could contribute to the assumed biomechanical support. However, further studies are needed to determine whether this pressure distribution translates into improved protective or clinical outcomes.

Conversely, the pressure distribution in the lower region (EL and SL) showed an opposite pattern, with 3DP exhibiting the least pressure and Medi-H the greatest. Particularly at the EL region, pressures measured during the flexion and pick-up tasks were 127% and 133% higher for Medi-H than for 3DP, respectively. This difference may be explained by the design of 3DP, which closely conforms to the spinal curvature in static posture but tends to tilt outward at the inferior edge during forward movements. Such behavior may reduce effective anchoring in the lower region, thereby decreasing contact pressure.

Whereas forward movement tasks primarily challenge anterior shear control, trunk flexion and extension increase posterior contact demands and loading on the paraspinals. During the extension task, pressure was primarily concentrated at the superior and inferior ends of all braces. Notably, 3DP generated an average pressure 54% greater across all regions compared with Medi-S, indicating enhanced support. Similar findings have been reported by Terai et al. [22]; the study demonstrated that the pressure during trunk extension was also mainly distributed at the superior and inferior ends of the braces, with the custom-made stay brace exhibiting mean pressure values approximately 60% greater than those of the aluminum-stayed brace and nearly double those of the plastic-stayed brace. Together, these findings further support the role of customized spinal braces in providing effective stabilization during trunk extension.

In addition, the observed pressure patterns further highlight the structural differences between the three braces. 3DP generated greater pressures in the erector spinae regions (EU and EL), whereas greater pressures in the spinal vertebral regions (SU and SL) were observed with Medi-H. The increased paraspinal pressures associated with 3DP are likely attributable to its thicker 3D-printed structure along the erector spinae, which provides enhanced supportive force during trunk extension. In contrast, the elevated pressures observed with Medi-H over the spinal regions (SU and SL), along with a localized peak at the EM region, are likely the result of its rigid external plastic panel and strap configuration, which applies direct contact forces to these areas.

Based on the present findings, the 3DP brace demonstrated superior overall supportive performance compared with traditional braces. The advantages of the 3DP were attributed to the semi-rigid 3D printing material and semi-custom-made orthosis. However, reduced pressure patterns were observed at the lower region during the flexion and pick-up tasks, where 3DP tended to tilt outward and exhibit reduced contact pressure. To address this limitation, future design modifications could consider optimizing the strap configuration at the lower region to enhance direct contact pressure. Such adjustments may improve lower-region stabilization, which is similar to the localized pressure effect observed at the EM region of Medi-H.

### 4.2. ROM Data

The three spinal segments selected (C7, T8, and L3) represent the apex of normal lumbar curvature and have also been commonly used to evaluate spinal ROM in previous studies [11,17]. Overall, the thoracic and lumbar ROM results under different tasks were roughly similar and with no statistically significant difference across the three braces, indicating that they could effectively limit individuals to a comparable extent. The best limitation in the flexion task was 3DP (29.64°), the extension task was Medi-H (−22.59°), and the pick-up task was Medi-S (23.47°).

When considered alongside previous studies, the present results indicate that customized spinal braces generally provide greater motion restriction than standardized designs, and that this effect is closely associated with the material stiffness of aluminum and plastic [22,27]. Lang et al. [28] reported that customized rigid and semi-rigid braces achieved greater ROM restriction than a traditional brace. Similar findings were reported by Terai et al. [22], who demonstrated that extension ROM restriction decreased from custom-made braces (−27.5°) to 3DP (−32.66°), aluminum-stayed braces (−33.4°), and plastic-stayed braces (−34.3°). Consistent with these previous studies, the present findings showed that braces with stiffer structural components tended to exhibit greater ROM restriction during controlled flexion and extension tasks when appropriately fitted to the individual’s trunk. The semi-rigid material of the 3DP was suitable for use in the spinal brace, as it provided support and limited ROM.

Notably, the pick-up task showed a different pattern, with Medi-S imposing the greatest restriction (23.47°), followed by 3DP (24.16°), then Medi-H (27.55°). This observation aligns with Fercho et al. [11]. The Hohmann brace, characterized by a lesser rigidity, has the least lumbar inclination angle (1.3°), indicating that it could maintain the posture in a relatively upright position. These results confirm that for complex movements, ROM restriction may not be entirely due to brace stiffness but rather primarily arises from muscle responses induced by the biofeedback mechanism and intra-abdominal pressure. A softer brace may be more effective in this regard.

### 4.3. Limitation

Overall, the novelty of this work lies in its rapid manufacturing protocol, which significantly reduces production lead time and costs compared to fully customized alternatives. Beyond cost-efficiency, the semi-customized design achieves superior anatomical conformity and enhanced structural support relative to traditional braces. Furthermore, this study’s results provide biomechanical evidence by simultaneously mapping regional interface pressures and trunk kinematics during multi-directional functional tasks. However, the study was performed under some limitations. (1) This study focused on the effectiveness of the design of the spinal brace. To avoid causing a second injury to the patient with low back pain, the study population was limited to healthy volunteers rather than clinical patients, which may affect the generalizability of the results to individuals with spinal pathologies. (2) A skewed gender ratio may have introduced biological or anatomical biases into the study. (3) Each brace was fitted and adjusted to ensure it was within a comfortable and acceptable tolerance range for every participant, avoiding localized pain or tissue pinching. However, subjective comfort scores (e.g., VAS, visual analog scale for tightness) were not formally quantified in this study. (4) Although anatomical landmarks (such as spinous processes and erector spinae muscle) were manually palpated and used for initial sensor alignment, dynamic skin deformation and minor relative displacement between the brace and skin could introduce spatial alignment uncertainty. Consequently, the regional pressure profiles reported herein represent overall contact mechanics across defined surface projection zones, rather than tissue-specific measurements of individual spinal segments.

### 4.4. Future Work

(1) The sample size was relatively small and consisted primarily of a specific cohort, which may limit the generalizability of the findings. To enhance the reliability and overall validity of these biomechanical evaluations, future studies should recruit a larger, more diverse participant population, including individuals across age groups and with varying clinical conditions. (2) The research was cross-sectional in nature; therefore, the long-term physiological effects and durability of the 3DP device could be studied in the future. (3) Since mesh density and filament dimensions in the 3D-printed brace critically dictate local brace stiffness and lumbar support performance, the Taguchi method or finite element method with optimization will be employed in future designs. This systematic optimization framework will help determine the ideal combination of structural mesh parameters to maximize spinal stability while enhancing wearer comfort. (4) Recently, 4D printing of magneto-responsive shape memory polymers (MSSMPs) offers revolutionary potential in assistive, rehabilitation, and wearable technologies through material change driven by external temperature [29]. 4D-printed MSSMP spinal braces advance 3D-printed orthoses by replacing static mesh structures with dynamic, stimulus-driven adaptability. This adjustable material allows the brace to soften for correction and comfort while maintaining motion restriction comparable to conventional rigid braces [30]. In future studies, this integration of MSSMPs should enhance breathability and optimize anatomical stabilization of the spinal brace.

## 5. Conclusions

This study demonstrates that semi-customized 3DP effectively achieved sagittal ROM restriction comparable to traditional commercial braces (Medi-H and Medi-S), while delivering superior, targeted interface pressure distribution across key anatomical regions. Specifically, across static standing, trunk flexion, trunk extension, and pick-up tasks, all three braces maintained thoracic and lumbar motion restrictions within similar functional thresholds; however, the 3DP brace provided significantly greater structural support to the upper and middle lumbar spine as well as the erector spinae musculature compared to traditional designs.

Crucially, high-support options like Medi-H generated localized pressure concentrations on the spinal column during extension. In contrast, the 3DP brace successfully redistributed contact forces toward the paraspinal musculature, thereby reducing direct pressure on bony prominences without compromising overall stability. From a clinical perspective, the ability of 3DP braces to deliver targeted regional unloading suggests that personalized additive manufacturing can directly mitigate common adverse effects associated with conventional braces.

## Figures and Tables

**Table 1:** Thoracic and lumbar ROM in the sagittal plane.

		3DP	Medi-H	Medi-S
Flexion	C7-T8	24.86 (11.53)	27.85 (14.01)	29.12 (15.06)
	T8-L3	29.64 (2.73)	34.07 (2.28)	30.28 (3.44)
Extension	C7-T8	−23.60 (10.79)	−22.42 (10.08)	−23.36 (10.92)
	T8-L3	−32.66 (22.8)	−22.59 (11.73)	−39.22 (18.97)
Pick up	C7-T8	22.28 (13.37)	29.34 (21.51)	21.48 (9.99)
	T8-L3	24.16 (11.23)	27.55 (11.17)	23.47 (11.49)

Note: Mean (SD): positive values mean forward movement; negative values mean backward movement.

## Data Availability

The original contributions presented in the study are included in the article; further inquiries can be directed to the corresponding author.
